# Extremely low flow tracheal gas insufflation of helium-oxygen mixture improves gas exchange in a rabbit model of piston-type high-frequency oscillatory ventilation

**DOI:** 10.1186/1475-925X-12-29

**Published:** 2013-04-08

**Authors:** Atsushi Baba, Tomohiko Nakamura, Tetsuya Aikawa, Kenichi Koike

**Affiliations:** 1 Department of Pediatrics, Shinshu University School of Medicine, Matsumoto, Nagano, 390-8621, Japan; 2 Division of Neonatology, Nagano Children’s Hospital, Toyoshina, Azumino City, Nagano, 399-8288, Japan; 3 First Development Group, Corporate Research Division, Air Water R&D Co., Ltd, Matsumoto, Nagano, 390-1701, Japan

## Abstract

**Objective:**

The purpose of this study was to show the effects of the tracheal gas insufflation (TGI) technique on gas exchange using helium-oxygen mixtures during high-frequency oscillatory ventilation (HFOV). We hypothesized that a helium-oxygen mixture delivered into the trachea using the TGI technique (0.3 L/min) would enhance gas exchange during HFOV.

**Methods:**

Three rabbits were prepared and ventilated by HFOV with carrier 70% helium/oxygen or 70% nitrogen/oxygen gas mixture with TGI in a crossover study. Changing the gas mixture from nitrogen70% to helium70% and back was performed three times per animal with constant ventilation parameters.

**Results:**

Compared with the nitrogen-oxygen mixture, the helium-oxygen mixture of TGI reduced PaCO_2_ by 7.6 mmHg (p < 0.01) and improved PaO_2_ by 14 mmHg (p < 0.01). Amplitude during TGI was significantly lower with the helium-oxygen mixture than with the nitrogen-oxygen mixture (p < 0.01) and did not significantly affect mean airway pressure.

**Conclusions:**

This study demonstrated that a helium-oxygen mixture delivered into the trachea using the TGI technique would enhance CO_2_ elimination and improve oxygenation during HFOV.

## Background

In spite of advances in approach and therapeutic benefits of conventional mechanical ventilation in respiratory failure in the neonatal intensive care unit (NICU), ventilator-induced lung injury remains a major problem. This is particularly relevant in patients who need aggressive maintenance of pressures and FiO_2_ for adequate oxygenation. In such patients, high-frequency oscillatory ventilation (HFOV) has been considered advantageous for maintaining oxygenation using higher mean airway pressure with minimal risk of complication. During HFOV, tidal volume (Vt) and associated swings in alveolar pressure are very small [1,2]. HFOV has been used in a variety of clinical situations, including neonatal respiratory distress syndrome (RDS), congenital diaphragmatic hernia (CHD), meconial aspiration syndrome (MAS), air leak syndrome and other [3-5]. HFOV showed a number of different mechanisms in addition to bulk convection that have been postulated to account for gas exchange, including Taylor dispersion and turbulence, asymmetric velocity profiles, pendelluft, cardiogenic mixing, collateral ventilation and molecular diffusion [6].

Helium is a noble gas, with very low atomic weight (4 g/mol) and density (0.157 g/L at 37°C and 1 atm). The density of helium-oxygen mixture reduces the resistance factor in gas delivery [7]. This increased mobility has three effects: gas more readily reaches the alveoli, thus allowing greater diffusion; breathing effort is significantly reduced by using a less-dense gas; and carbon dioxide is eliminated more rapidly through a helium-oxygen mixture than through a nitrogen-oxygen mixture [8-10]. In HFOV with helium, both properties may contribute to improved ventilation [11]. Winters et al. reported a case series of children with respiratory acidosis during HFOV. When the carrier gas was changed to a helium-oxygen mixture, CO_2_ clearance improved [12]. Helium-oxygen mixtures have been examined using HFOV in animal models [9,10,13]. Those experiments showed that the improvement of ventilation with a helium-oxygen mixture was related to a larger Vt delivery by the oscillator under the same amplitude (AMP). If Vt during HFOV is maintained at a constant level, use of a helium-oxygen mixture does not alter gas exchange in HFOV ventilators with either a membrane-driven oscillator [10] or a piston-driven oscillator [13].

Continuous tracheal gas insufflation (TGI) is a technique that flushes dead space, and may thus allow reductions in respiratory support. Kakous et al. [14] reported the effects of continuous TGI in conventional ventilation. The effect of TGI is based mainly on the replacement of end-tidal gas in the instrumental dead space with an inspiratory gas mixture.

We hypothesized that a helium-oxygen mixture delivered into the trachea using a TGI technique (0.3 L/min) would enhance CO_2_ elimination during HFOV. The purpose of this study was to compare the effects of TGI on gas exchange using helium-oxygen and nitrogen-oxygen mixtures in a rabbit model during HFOV.

## Methods

### Bench test

**Figure 1:**
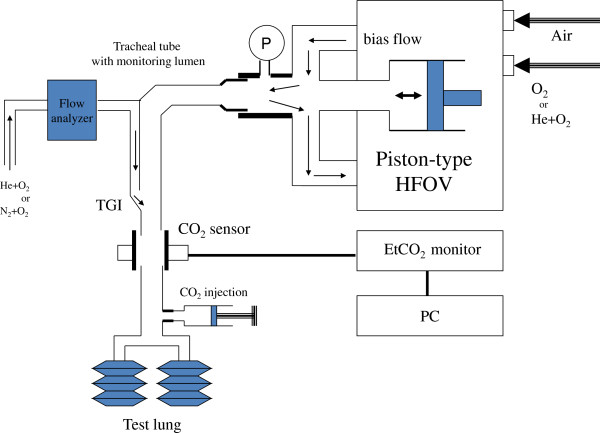
**Schematic of the experimental system.** The carrier gas (nitrogen-oxygen mixture or helium-oxygen mixture; bias flow rate, 8 L/min) inside the HFOV circuit was oscillated with mean airway pressure set at 12 cmH_2_O, stroke volume at 15 mL, and frequency of 15 Hz. Helium-oxygen mixture was supplied at the O_2_ port of the ventilator. In the series of HFOV with TGI, insufflating gas flow (nitrogen-oxygen mixture or helium-oxygen mixture) through the monitoring lumen of the ETT was 0.3 L/min.

To elucidate the physical effects of reducing gas density during HFOV with TGI, we performed a bench test utilizing a test lung. A schematic of the experimental system is shown in Figure 1. The test lung (NeoLung; IngMar Medical, PA) was connected to the endotracheal tube (ETT) with a monitoring lumen (internal diameter, 3.5 mm; Mallinckrodt, St. Louis, MO), and attached to the circuit of a piston-driven high-frequency ventilator (Humming II; Metran, Saitama, Japan). Static compliance and resistance of the test lung were1.3 mL/cmH_2_O/kg and 160 cmH_2_O/L/s/kg, respectively. Pressure at the Y-piece of the circuit was monitored using a pressure sensor (AP-C35; Keyence, Osaka, Japan). The carrier gas (nitrogen-oxygen or helium-oxygen mixture, 78:22 mixture ratio; bias flow rate, 8 L/min) inside the HFOV circuit was oscillated using the piston-driven Humming II with mean airway pressure set at 12 cmH_2_O, stroke volume (SV) at 15 mL, and frequency at 15 Hz.

In the series of HFOV with TGI, the carrier gas inside the HFOV circuit was a nitrogen-oxygen mixture (78:22 mixture ratio). Insufflating gas flow (nitrogen-oxygen mixture, 78:22 mixture ratio; or helium-oxygen mixture, 78:22 mixture ratio) through the monitoring lumen of the ETT was 0.3 L/min. We used a helium-calibrated flow analyzer (PF-300; ImtMedical, Buchs, Switzerland) to ensure that TGI flow of the two gases was equal. To compare the speed of CO_2_ transport, 5 ml of tracer gas (CO_2_) was injected into the system through an access port near the test lung. CO_2_ partial pressure at the end of the ETT was monitored using an EtCO_2_ monitor (OLG-2800; Nihon Kohden, Tokyo, Japan). Real-time data from the EtCO_2_ monitor were collected using a computerized data accumulation system at a sampling rate of 200 samples/s. The single exponential curve describing the CO_2_ concentration inside the system for 3 s was approximated by nonlinear regression (Excel 2010; Microsoft, WA). We used the equation: measured CO_2_ concentration = *a* exp^-*b t*^, where *a* and *b* are the variables obtained by curve fitting and *t* is the time of CO_2_ measurement from the point at which CO_2_ concentration was 100 mmHg, representing the upper limit of the measurement capability of the EtCO_2_ monitor. All experiments were repeated six times.

### Animal preparation

All study protocols were approved by the Institutional Animal Care and Use Committee of Shinshu University, Nagano, Japan. Three Japanese white rabbits (body weight, 2.309 ± 0.131 kg) were used in the study. Animals were premedicated by intramuscular administration of midazolam (10 mg/kg/dose) and xylazine (5 mg/kg/dose). The ear vein was cannulated using a 24-G angiocatheter for intravenous anesthesia and hydration. Animals were placed in a supine position under a radiant warmer to maintain body temperature throughout the entire study period, and body temperature was Tmonitored using a rectal temperature probe. Tracheotomy was performed, and a no-cuff ETT with monitoring lumen (internal diameter, 3.5 mm; Mallinckrodt), was inserted to a depth of 3 cm from the lower edge of the cricoid cartilage and fixed in place. Intermittent ventilation (IMV) was initiated using a time-cycled, pressure-limited ventilator (Humming II; Metran). Using a digital pressure sensor (AP-C40; Keyence) installed into the Y-piece of the breathing circuit, actual pressure parameters were measured and registered.

Static compliance and resistance were measured by the passive expiratory flow-volume method, using a pneumotachograph (LFM-317 Aivision Laminar Flow Meter, Metabo, Lausanne, Switzerland). During this procedure, we confirmed that no leakage was present. Anesthesia and myoparalysis were provided by continuous intravenous infusion of midazolam (0.1 mg/kg/h), xylazine (3 mg/kg/h) and pancuronium (0.1 mg/kg/h). The carotid artery was then cannulated for direct blood pressure measurement, heart rate (HR) monitoring and determination of arterial blood gases (ABG).

### Interventions and measurements

Animals were allowed 20 min for stabilization under IMV. After sustained inflation maneuver at 20 cmH_2_O for 10 s, ventilation mode was shifted to the HFOV mode of the same ventilator (Humming II; Metran). ABG was obtained after 10 min of ventilation with: mean airway pressure (MAP), 12 cmH_2_O; frequency, 15 Hz; FiO_2_, 0.30; and bias flow rate, 8 L/min. SV (same as Vt) was adjusted to 4.16 ± 0.57 ml (mean ± standard deviation) to obtain permissive hypercapnia. The target PaCO_2_ was 70 mmHg. Amplitude (AMP) was a subordinate factor during HFOV with the piston-driven oscillator, which was influenced by SV, animal airway condition (especially compliance) and the nature of the gas in the circuit. After baseline recordings were taken, tracheal gas was insufflated through the second monitoring lumen of the ETT for 10 min at gas flow of 0.3 L/min. In our preliminary experiment in healthy rabbits, steady state was reached after approximately 5 min during HFOV with TGI. After data collection with a nitrogen-oxygen mixture (70:30 mixture ratio), the gas for TGI was switched to a helium-oxygen mixture (70:30 mixture ratio, 0.487 g/L at 37°C and 1 atm) and ventilated for 10 min before obtaining data. After data collection with the helium-oxygen mixture (70:30), the gas for TGI was returned to the nitrogen-oxygen mixture (70:30), and allowed 10 min to stabilize before data collection. HFOV settings were held constant while the gas mixture of TGI was changed. The cycle of changing the gas mixture from nitrogen-oxygen mixture to helium-oxygen mixture and back was performed three times per animal. To ensure that animal conditions returned to baseline after each experiment, rabbits were ventilated under HFOV without TGA.

### Statistics

Data were analyzed by Wilcoxon *t*-test, one-way analysis of variance (ANOVA) or repeated-measures ANOVA, followed by the Bonferroni test to compare matched experimental sets. All data are presented as mean ± standard error of the mean. Significance was defined for values of p < 0.01.

## Results

### Bench test

**Figure 2:**
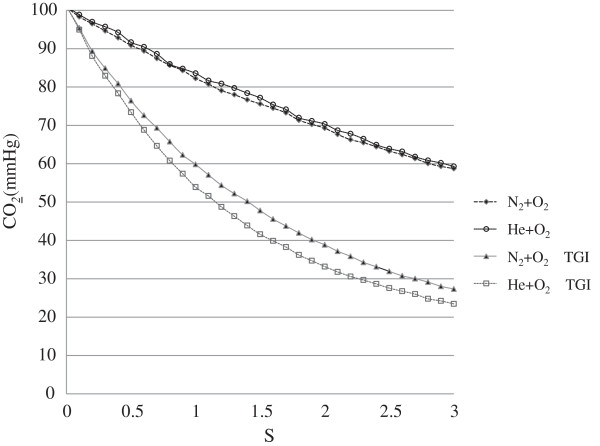
**CO_2_ elimination curve.** The average of CO_2_ elimination in comparison of nitrogen-oxygen and helium-oxygen mixtures with or without TGI.

The CO_2_ elimination curve is shown in Figure 2. Speed of CO_2_ transport is expressed as the time for the CO_2_ concentration to reach 63% of the final concentration (time constant). Effects of the speed of CO_2_ transport on the time constant are summarized in Table 1. When the carrier gas inside the HFOV circuit was changed without TGI, the time constant was 5.54 ± 0.26 s with the nitrogen-oxygen mixture and 5.57 ± 0.06 s with the helium-oxygen mixture. When the gas for TGI was changed to a low-density gas, speed of CO_2_ transport increased, as reflected by a significant fall in time constant from 2.3 ± 0.01 s with the nitrogen-oxygen mixture to 2.05 ± 0.04 s with the helium-oxygen mixture (p < 0.01).

**Table 1:** Effects of the speed of CO2 transport on the time constant

**Insufflating gas**	**Non-linear regression**	**b**	**Time constant = 1/|b|**
Helium-oxygen	y = 91.641e^-0.4848x^	−0.4848	**2.06**
	y = 91.24e^-0.4986x^	−0.4986	**2.01**
	y = 89.045e^-0.4748x^	−0.4748	**2.11**
	y = 91.327e^-0.4936x^	−0.4936	**2.03**
	y = 93.579e^-0.4859x^	−0.4859	**2.06**
	y = 92.799e^-0.492x^	−0.492	**2.03**
			**mean ± SD 2.05 ± 0.04**
Nitrogen-oxygen	y = 94.984e^-0.436x^	−0.436	**2.29**
	y = 94.806e^-0.436x^	−0.436	**2.29**
	y = 94.334e^-0.436x^	−0.436	**2.29**
	y = 95.175e^-0.4367x^	−0.4367	**2.29**
	y = 93.381e^-0.4321x^	−0.4321	**2.31**
	y = 96.175e^-0.437x^	−0.437	**2.29**
			**mean ± SD 2.3 ± 0.01**
**Bias flow gas**	**Non-linear regression**	**b**	**Time constant = 1/|b|**
Helium-oxygen	y = 101.18e^-0.182x^	−0.182	**5.49**
	y = 100.9e^-0.181x^	−0.181	**5.52**
	y = 99.006e^-0.1774x^	−0.1774	**5.64**
	y = 100.35e^-0.1807x^	−0.1807	**5.53**
	y = 101.64e^-0.1797x^	−0.1797	**5.56**
	y = 99.601e^-0.1771x^	−0.1771	**5.65**
			**mean ± SD 5.57 ± 0.06**
Nitrogen-oxygen	y = 99.37e^-0.1925x^	−0.1925	**5.19**
	y = 98.925e^-0.1801x^	−0.1801	**5.55**
	y = 100.75e^-0.1751x^	−0.1751	**5.71**
	y = 99.517e^-0.1738x^	−0.1738	**5.75**
	y = 100.72e^-0.1733x^	−0.1733	**5.77**
	y = 96.836e^-0.1912x^	−0.1912	**5.23**
			**mean ± SD 5.54 ± 0.26**

### Animal experiments

**Figure 3:**
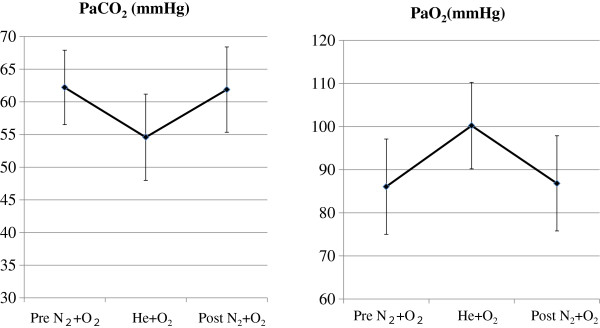
**PaCO_2_ and PaO_2_ under TGI.** The gas mixture under TGI was changed from nitrogen-oxygen to helium-oxygen and back.

We compared PaCO_2_ and PaO_2_ values obtained after 10 min of TGI with each of the two gas mixtures (Figure 3). Compared with the nitrogen-oxygen mixture, the helium-oxygen mixture reduced PaCO_2_ by 7.6 mmHg (p < 0.01) and improved PaO_2_ by 14 mmHg (p < 0.01). AMP during TGI was significantly lower with the helium-oxygen mixture than with the nitrogen-oxygen mixture (p < 0.01) and did not significantly impact MAP (Table 2).

**Table 2:** AMP and MAP change during the animal experiment

	**No TGI**	**TGI N**_**2**_ **+ O**_**2**_	**TGI He + O**_**2**_	**TGI N**_**2**_ **+ O**_**2**_	**TGI He + O**_**2**_	**TGI N**_**2**_** + O**_**2**_	**TGI He + O**_**2**_	**TGI N**_**2**_ **+ O**_**2**_	**No TGI**
Rabbit 1 AMP (cm H_2_O)	19	19.2	17.4^*^	19.5	17.7^*^	19.3	17.2^*^	19.2	19.8
Rabbit 2 AMP (cm H_2_O)	17.5	17.5	15.9^*^	17.5	16.1^*^	17.9	16^*^	17.6	17.8
Rabbit 3 AMP (cm H_2_O)	15.8	15.9	14.2^*^	16	14.4^*^	16	14.4^*^	16.1	16.3
Rabbit I MAP (cm H_2_O)	12	11.9	11.7	12	11.6	11.8	11.6	11.8	12
Rabbit 2 MAP (cm H_2_O)	12	12	12.1	12.2	12.1	12.2	12	12.1	12.1
Rabbit 3 MAP (cm H_2_O)	12.7	12.7	12.7	12.9	12.7	12.9	12.8	12.7	12.8

*AMP* amplitude, *MAP* mean air-way pressure, *TGI* tracheal gas insufflation.

*: p < 0.01.

## Discussion

Helium may alter gas exchange during HFOV via a number of mechanisms. Because helium is less dense than nitrogen, the frictional forces in turbulent flows are reduced with helium as compared to oxygen-enriched air. For a given set of airway dimensions, turbulent flow results in a higher resistance than laminar flow. In addition, mechanical ventilation through a narrow ETT and airway, particularly in pediatric and neonatal patients, may further increase the Reynolds number, thus resulting in greater turbulent flow [15,16]. With helium, the calculated Reynolds number is lower (<200), which may change regions of turbulent flow to laminar flow, reducing resistance and energy leakage. As resistive forces and energy dissipation are decreased, tidal volume per oscillation increases. This theory has been confirmed before in a laboratory study [8]. In theory, because of its lower density, helium may favorably alter gas exchange through the pendelluft effect, inhalation/exhalation flow asymmetry, Taylor dispersion, and molecular diffusion [9].

By flushing dead space, continuous TGI may allow reductions in respiratory support. The effect of continuous TGI is based mainly on the replacement of end-tidal gas in the instrumental dead space with an inspiratory gas mixture. Continuous TGI allows the use of low-volume ventilation over a prolonged period and reduces the duration of mechanical ventilation in preterm infants [17]. To facilitate CO_2_ transport without increasing SV and AMP, a helium-oxygen mixture was administered to the turbulent zone using TGI techniques.

When using a helium-oxygen mixture for TGI, CO_2_ excretion speed increases under constant SV. This effect is attributed to the nature of helium in the turbulent zone. A significant decrease in PaCO_2_ was shown in animal experiments performed to reproduce the effects apparent in the bench test. The concentration of helium in the test lung that resulted from mixing the TGI flow with the main flow was same as main flow. When we supplied 0.6 ml/min of TGI flow, helium-oxygen was not needed on the ventilator. We therefore propose that TGI offers a very simple, cost-effective means of supplying helium-oxygen during HFOV, without the need to adapt/calibrate the ventilator for the use with helium-oxygen and avoiding the high gas consumption associated with a high bias flow rate.

One limitation of the present study was that our experiment was performed in normal animal lungs. The low density of helium does not always reduce resistance [18]. Different types and phenotypes of obstructive airway disease manifest in different regions of the lung. The effectiveness of TGI on helium-oxygen mixture HFOV may thus differ according to the type of obstructive airway disease. Further research using lung injury models is therefore warranted.

## Conclusion

This study demonstrated that a helium-oxygen mixture delivered into the trachea using a tracheal gas insufflation (TGI) technique enhances CO_2_ elimination and offers a simple cost-effective means of achieving helium-oxygen mixture HFOV.

## Competing interests

The authors declare that they have no competing interests.

## Authors’ contributions

AB participated in the design of the study and performed the statistical analysis. TN conceived of the study, participated in its design and coordination and helped to draft the manuscript. TA participated in the design of the study and performed the statistical analysis. KK participated in the design of the study and performed the statistical analysis. All authors have read and approved the final manuscript.
